# Dietary Supplementation with 25-Hydroxyvitamin D_3_ on Reproductive Performance and Placental Oxidative Stress in Primiparous Sows during Mid-to-Late Gestation

**DOI:** 10.3390/antiox13091090

**Published:** 2024-09-06

**Authors:** Jing Li, Qingyue Bi, Yu Pi, Xianren Jiang, Yanpin Li, Xilong Li

**Affiliations:** 1 Key Laboratory of Feed Biotechnology of Ministry of Agriculture and Rural Affairs, Institute of Feed Research, Chinese Academy of Agricultural Sciences, Beijing 100081, China; lijingbj_87@126.com (J.L.); biqingyue981106@126.com (Q.B.); piyu@caas.cn (Y.P.); jiangxianren@caas.cn (X.J.); 2 College of Agriculture, Yanbian University, Yanji 133000, China

**Keywords:** 25 hydroxyvitamin D_3_, primiparous sows, reproductive performance, placenta, oxidative stress

## Abstract

The placenta plays a crucial role in nutrient transport and waste exchange between the dam and fetus, sustaining fetal growth. While the positive effects of 25-hydroxyvitamin D_3_ (25-OH-D_3_) on animal performance have been reported, its impact on placental function remains largely unknown. Therefore, this study aimed to investigate the effects of supplementing 25-OH-D_3_ in the diet of primiparous sows on reproductive performance, antioxidant capacity, placental oxidative stress, nutrient transport, and inflammatory response during mid-to-late gestation. A total of 45 healthy Landrace × Yorkshire primiparous sows on day 60 of gestation were selected and randomly allocated to three treatment groups based on body weight and backfat thickness: the control group (corn-soybean meal basal diet), the VD_3_ group (basal diet + 2000 IU VD_3_), and the 25-OH-D_3_ group (basal diet + 50 μg/kg 25-OH-D_3_). The results demonstrated that supplementation with 25-OH-D_3_ in the diet enhanced sows’ average litter weight and birth weight during mid-to-late gestation. Additionally, plasma malondialdehyde (MDA) concentrations in sows significantly decreased in the VD_3_ and 25-OH-D_3_ groups (*p* < 0.05). Furthermore, lower gene expressions of placental *HO-1*, *GPX2*, *IL-8*, and *IL-6* were found in the VD_3_ or 25-OH-D_3_ groups (*p* < 0.05 or *p* < 0.10), while higher gene expressions of *GLUT1* and *SNAT2* in the placenta of sows were observed in the VD_3_ and 25-OH-D_3_ groups, respectively (*p* < 0.05). These findings indicate that the supplementation of VD_3_ and 25-OH-D_3_ in the diet of sows can improve their plasma oxidative stress status, enhance placental antioxidant capacity and nutrient transport, and reduce placental inflammatory responses, with more pronounced improvements in sow performance observed in sows fed diets supplemented with 25-OH-D_3_.

## 1. Introduction

In commercial swine production, the reproductive performance of sows, which includes the number of piglets born and alive, birth weight, and litter weight, is the most crucial economic indicator closely associated with pig production efficiency [1]. It is wellknown that sow pregnancy is a dynamic and precise process involving embryonic development, physiological changes, fetal growth, placental development, and maternal body reconstruction, leading to increased metabolic demands on sows, especially during mid-to-late gestation [2,3]. Numerous studies have demonstrated that maternal metabolism significantly increases during late gestation to meet the rapid fetal growth’s energy and nutrient needs. However, this process can also lead to excessive reactive oxygen species (ROS) production, resulting in maternal oxidative stress, particularly for hyper-prolific sows, which can impact energy balance, reproductive performance of sows, and subsequent piglet livability [4,5,6]. Furthermore, there is evidence suggesting that maternal oxidative stress also impacts placental oxidative stress status and function, such as trophoblast proliferation and differentiation and angiogenesis, which can limit the nutrient delivery from the dam to the fetus, ultimately affecting fetal metabolism and development [7,8,9].

Vitamin D_3_ (VD_3_) is a critical fat-soluble vitamin with a unique absorption mechanism that involves two hydroxylation processes. It is converted to the active form, 25-hydroxyvitamin D_3_ (25-OH-D_3_), via 25-hydroxylase (CYP24A1) in the liver and then converted to 1,25-dihydroxy vitamin D_3_ (1,25-(OH)_2_-D_3_) by 1 α-hydroxylase (CYP27B1) in the kidney [10]. VD_3_ participates in multiple biological processes, including intestinal calcium and phosphorus absorption, bone and muscle development, cell proliferation and differentiation, and the regulation of oxidative stress and inflammation [11,12,13]. In recent years, some reports revealed that exposure to oxidative stress affects the absorption of VD_3_ and inhibits its conversion to 25-OH-D_3_ in the liver. In contrast, dietary supplementation of 25-OH-D_3_ could enhance maternal antioxidant capacity and improve the VD_3_ status of the mother and fetus during the late gestation and lactation periods of sows [14,15,16]. However, there is a lack of knowledge regarding the dietary supplementation of 25-OH-D_3_ in alleviating placental oxidative stress and its effects on nutrient transfer and fetal development in sows. Therefore, the present study aims to investigate the effects of the active and original forms of VD_3_ on the reproductive performance, placenta antioxidant capacity, and function of primiparous sows via supplementing equivalent doses of VD_3_ and 25-OH-D_3_ to the diet during mid-to-late gestation.

## 2. Materials and Methods

### 2.1. Ethic Statement

The animal experiment was conducted at the Shandong Liaocheng Experimental Pig Farm, and all the procedures in this study were performed following the Institutional Animal Care and Use Committee of the Institute of Feed Research of the Chinese Academy of Agricultural Sciences (IFR-CAAS-20230908).

### 2.2. Animals and Experimental Design

On day 60 of gestation, a total of 45 healthy Landrace × Yorkshire primiparous sows were selected and randomly allocated into 3 groups based on body weight (198.3 ± 10.2 kg) and backfat thickness (18.85 ± 0.44 mm). Each group had 15 replicates, with 1 sow per replicate. The control group (CT) was fed the commercial gestation diet, the VD_3_ group was fed the CT diet supplemented with 2000 IU/kg VD_3_, and the 25-OH-D_3_ group was fed the CT diet supplemented with 50 µg/kg 25-OH-D_3_, whose active substance equaled to 2000 IU/kg VD_3_. The products of cholecalciferol (>99% VD_3_) and calcifediol monohydrate (>98% 25-OH-D_3_) were provided by Shandong Tonghui Biotechnology Co., Ltd. (Binzhou, China). The commercial gestation diet for sows was formulated according to the National Research Council’s (NRC, 2012) nutritional requirements (shown in Table 1). The entire experimental period spanned 54 d, from day 60 of gestation to the end of farrowing.

On day 60 of gestation, the sows were housed in the same room and fed 2.5 kg of experimental diet per day (from day 60 to day 85 of gestation) and 3.0 kg of experimental diet per day (from day 85 of gestation to farrowing), with equivalent feedings at 8:00 am and 4:00 pm. On day 107 of gestation, each sow was transferred to a separate farrowing crate (2.20 m × 1.80 m) following the pre-farrowing feeding procedure. The gestation and farrowing rooms were maintained at 22 °C and 25 °C, respectively. All the sows were managed in accordance with routine immunization and deworming procedures.

### 2.3. Reproductive Performance Analysis

The body weight and backfat thickness of sows were measured on days 60 and 107 of gestation. Backfat thickness was measured using ultrasound equipment (Renco Lean-Meatier; Renco Corporation, Manchester, MA, USA) at a point 65 mm to the right of the dorsal midline of the last rib. At farrowing, the number of total piglets born, born alive, stillborn, mummies, and piglets weighing less than 1.0 kg were recorded for each sow. The piglets were weighed within 24 h after farrowing, and the average birth weight and average litter weight were calculated.

### 2.4. Sample Collection

On days 60 and 107 of gestation, 10 mL of blood was collected from the ear vein of each sow using a heparin tube. At farrowing, 10 mL of umbilical cord blood was extracted by pressing the umbilical cord blood from maternal side to fetus at a point 10–15 cm from the root. From each sow, 3 umbilical cord blood samples were collected from 3 different fetuses. All blood samples were centrifuged at 3000 rpm at 4 °C for 15 min to obtain plasma and stored at −20 °C for antioxidant indicator analysis.

In addition, a total of 3 placental tissues from each sow were immediately separated and placed in liquid nitrogen after farrowing. All samples were ground and crushed under liquid nitrogen conditions, transferred to the 2 mL cryopreservation tubes, and stored at −80 °C for further use.

### 2.5. Assay of Antioxidant Indicators

The antioxidant indicators in plasma and umbilical cord blood were determined, which included measuring the concentrations of malondialdehyde (MDA) and the activities of glutathione peroxidase (GSH-Px), superoxide dismutase (SOD), and catalase (CAT). The determination of the antioxidant indicators strictly followed the protocols of the individual ELISA kit specifically designed for pigs (Nanjing Jiancheng Bioengineering Institute, Nanjing, China).

### 2.6. Gene Expression Analysis

The total RNA was extracted from placental tissues using TRIzol reagent with an A260/280 value ranging between 1.8 and 2.0 and subsequently frozen at −80 °C until further use. The cDNA was obtained using the TakaRa reverse transcription kit (PrimeScriptTM RT reagent Kit with gDNA Eraser; Takara; Beijing, China). A quantitative real-time PCR (qRT-PCR) protocol was developed to measure the mRNA expression levels of antioxidant genes (*HO-1*, *Nrf2*, *SOD1*, *SOD2*, *CAT*, *GPX1*, and *GPX2*), nutrient transporter genes (*GLUT1*, *SNAT1*, *SNAT2*, and *VEGFA*), and inflammation genes (*IL-8*, *IL-6*, *IL-1β*, and *TNF-α*) according to the manufacturer’s instructions (TB Green^®^ *Premix Ex Taq* ™; Takara; Beijing, China). The cycling process of qRT-PCR was as follows: 95 °C for 3 min, 40 cycles of denaturing at 95 °C for 10 s and annealing at 60 °C for 30 s, and the final elongation at 60 °C for 5 s and ending at 95 °C for 10 s. The obtained data were analyzed to calculate the expression of target genes relative to GAPDH as a housekeeping gene using the 2-∆∆Ct method described by Livak et al. [17]. All primer sequences for the genes and GADPH (as a housekeeping gene) were designed by Primer 3.0, and the primers’ quality was tested using agarose gel electrophoresis. All primer sequences used in this study are listed in Table 2.

### 2.7. Statistical Analysis

All experimental data were analyzed using the one-way ANOVA in SPSS 22.0. The Tukey method was performed for multiple comparisons. All the data were considered a significant difference among treatments when *p* < 0.05 and a trend difference among treatments when 0.05 ≤ *p* < 0.10.

## 3. Results

### 3.1. Reproductive Performance

The effect of dietary supplementation with VD_3_ and 25-OH-D_3_ on the reproductive performance of primiparous sows is presented in Table 3. The results showed that there were no differences in sows’ body weight and average backfat thickness on days 60 and 107 among the three treatments (*p* > 0.05). Additionally, no differences were obtained in the number of total born, born alive, stillborn, and mummified among the treatments. Furthermore, the average litter weight and average piglet weight were higher in the 25-OH-D_3_ group, showing a significant increase compared to the VD_3_ group (*p* < 0.05). Moreover, it was found that the proportions of piglets with a birth weight less than 1.0 kg were lower in sows fed diets supplemented with VD_3_ and 25-OH-D_3_ during the mid-to-late gestation period compared to the CT group.

### 3.2. Plasma Antioxidant Capacities of Sows

The effect of dietary supplementation with VD_3_ and 25-OH-D_3_ on the plasma antioxidant capacity of primiparous sows is presented in Table 4. The results demonstrated that there were no significant differences in the concentrations of plasma MDA and the activities of SOD, GSH-Px, and CAT among the treatment groups on day 60 of gestation (*p* > 0.05). However, on day 107 of gestation, sows fed diets supplemented with VD_3_ and 25-OH-D_3_ reduced the concentration of plasma MDA (*p* < 0.05), whereas no significant impact was found on the activities of plasma SOD, GSH-Px, and CAT (*p* > 0.05).

### 3.3. Placenta Antioxidant Capacity

To further investigate the effects of dietary supplementation with VD_3_ and 25-OH-D_3_ on placental antioxidant capacity, we determined the antioxidant indicators in umbilical cord blood and the mRNA expression of the key antioxidant-related genes in placental tissue. The results revealed that there were no significant differences in the concentrations of MDA and the activities of SOD, GSH-Px, and CAT in the umbilical cord blood among the treatment groups (*p* > 0.05) (shown in Table 5).

**Figure 1:**
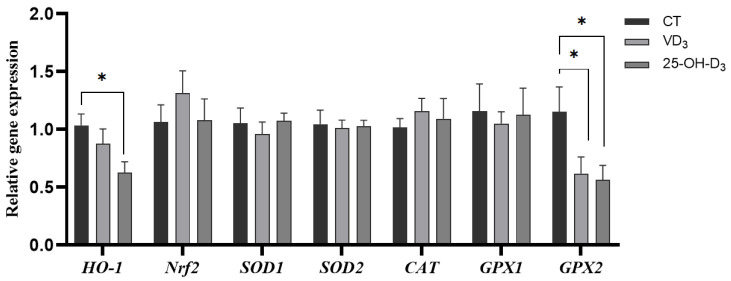
The effect of supplementing VD_3_ and 25-OH-D_3_ to the sow diet on the mRNA expression of placental antioxidant-related genes during mid-to-late gestation. The results are presented as mean ± SEM, n = 8. * represents a significant difference (*p* < 0.05). CT = control group fed with the basal diet; VD_3_ = VD_3_ group fed with the supplementation of 2000 IU/kg Vitamin D_3_ in the basal diet; 25-OH-D_3_ = 25-OH-D_3_ group fed with the supplementation of 50 µg/kg 25-OH-D_3_ in the basal diet. HO-1 = heme oxygenase-1; Nrf2 = nuclear factor-erythroid 2-related factor 2; SOD1 = superoxide dismutase 1; SOD2 = superoxide dismutase 2; CAT = catalase; GPX1 = glutathione peroxidase 1; GPX2 = glutathione peroxidase 2.

Furthermore, the gene expressions of placental HO-1 and GPX2 as the main antioxidant enzyme genes were significantly reduced in sows fed diet supplemented with 25-OH-D_3_ during the mid-to-late gestation, and the gene expression of GPX2 also decreased in the VD_3_ group compared to the CT group (*p* < 0.05). This indicated a lower oxidative damage response during nutrient transportation between sows and fetuses in the 25-OH-D_3_ and VD_3_ groups (shown in Figure 1).

### 3.4. Placental Function

**Figure 2:**
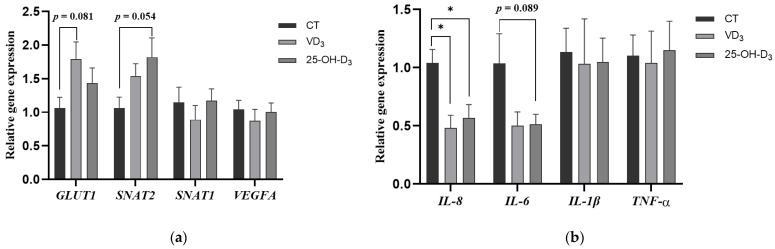
The effect of supplementing VD_3_ and 25-OH-D_3_ into the sow diet on the mRNA expression of placental nutrient transport (**a**) and immune-related genes (**b**) during mid-to-late gestation. The results are presented as mean ± SEM, *n* = 8. * represents a significant difference (*p* < 0.05). CT = control group fed with the basal diet; VD_3_ = VD_3_ group fed with the supplementation of 2000 IU/kg Vitamin D_3_ in the basal diet; 25-OH-D_3_ = 25-OH-D_3_ group fed with the supplementation of 50 µg/kg 25-OH-D_3_ in the basal diet. *GLUT1* = glucose transporter type 1; *SNAT2* = sodium coupled neutral amino acid transporter 2; *SNAT1* = sodium coupled neutral amino acid transporter 1; *VEGFA* = vascular endothelial growth factor A; *IL-8* = interleukin-8; *IL-6* = interleukin-6; *IL-1β* = interleukin-1β; *TNF-α* = tumor necrosis factor-α.

To further evaluate the effect of dietary supplementation with VD_3_ and 25-OH-D_3_ on placental function, we determined the gene expression of nutrient transport and immune-related genes in placental tissues. It was shown that the gene expression of glucose transporter GLUT1 in the placenta of sows fed the diet supplemented with VD_3_ tended to increase compared to the CT group (*p* = 0.081). Similarly, the gene expression of the neutral amino acid transporter SNAT2 in placental tissue from the 25-OH-D_3_ group showed a tendency toward upregulation compared to the CT group (*p* = 0.054). However, no significant differences were observed in the gene expression levels of SNAT1 and VEGFA in the placenta among the treatment groups (*p* > 0.05) (shown in Figure 2a).

In addition, the gene expression of pro-inflammatory factor IL-8 in the placenta of sows fed diets supplemented with VD_3_ and 25-OH-D_3_ was significantly decreased compared to the CT group (*p* < 0.05), and the gene expression of placental IL-6 in the 25-OH-D_3_ group tended to be reduced compared to the CT group (*p* = 0.089) (shown in Figure 2b).

## 4. Discussion

Sow reproductive performance plays a crucial role in pig production. Generally, sow reproduction relies on key performance indicators, primarily including litter size, litter weight, the number of born alive and stillborn piglets, etc [18]. Our study indicated that dietary supplementation with 25-OH-D_3_ enhanced the average litter weight and average piglet weight of primiparous sows during mid-to-late gestation, which was consistent with previous findings in multiparous sows [19,20]. Some researchers have reported that the supplementation of 25-OH-D_3_ to the diet of primiparous sows also increased the number of live-born piglets and the weaned litter size [15,21], but no significant improvement in the number of live-born piglets was observed in our findings. However, another study demonstrated that there was no significant effect on the reproductive performance of multiparous sows when fed diets supplemented with different doses of VD_3_ and 25-OH-D_3_ during early gestation, although high doses of VD_3_ and 25-OH-D_3_ could significantly reduce the number of stillbirths [22]. Furthermore, our study revealed no difference in sows’ performance between the CT group and the VD_3_ group, indicating that supplementing the sow diet with the same dose of 25-OH-D_3_ may be more efficient in improving the reproductive performance of sows during mid-to-late gestation.

Previous studies have demonstrated that the supplementation of 25-OH-D_3_ in the diet could alleviate oxidative stress and inflammatory responses through increasing plasma SOD and GSH-Px activity and reducing tumor necrosis factor (TNF)-α levels when animals were challenged with lipopolysaccharide (LPS) or sulfate colistin [23,24]. It is well known that sows suffer oxidative stress due to multiple factors, including increased metabolism, rapid fetal development, as well as the processes of parturition and milk production during gestation and lactation. Previous research has indicated that 25-OH-D_3_ could increase the antioxidant capacity of pregnant sows and their offspring [25], and high doses of VD_3_ have also been shown to improve the activities of antioxidant enzymes (GSH-Px, T-AOC, and T-SOD) and reduce MDA concentrations [26]. Consistent with these findings, the present study suggests that the same dose of 25-OH-D_3_ and VD_3_ can significantly reduce plasma MDA concentrations in primiparous sows and enhance maternal antioxidant capacity during late gestation.

In mammals, the placenta serves as a unique connecting structure between the mother and fetus, delivering the nutrients and waste to support fetal development via the umbilical cord. However, placental function can be impacted by maternal oxidative stress, ultimately reflecting the health status of both maternal and fetal growth [27]. A previous study has shown that an increase in maternal antioxidant capacity could enhance the antioxidant function of umbilical cord blood [28]. Although it did not show a significant improvement in the antioxidant indicators of the umbilical cord in the 25-OH-D_3_ group, the level of GSH-Px was numerically higher than that in other groups. In addition, some researchers reported that the gene expressions of placental *SOD*, *GSX,* and *CAT* were upregulated, and the livability of piglets and reproductive performance of sows were reduced when the maternal oxidative stress increased, indicating that the placenta initiated a compensatory mechanism by activating the Nrf2 signaling pathway to alleviate placenta damage induced by oxidative stress and maintain fetal growth [29,30,31,32]. In the present study, our findings illustrated that the supplementation with 25-OH-D_3_ in the sow diet downregulated the gene expressions of *HO-1* and *GPX2*. Additionally, the gene expression of *GPX2* decreased in the VD_3_ group compared to that in the CT group, suggesting both 25-OH-D_3_ and VD_3_ can alleviate the placental compensatory effects caused by oxidative stress and improve placental antioxidant capacity.

Placental nutrient transporters, including those for glucose, amino acids, and fatty acids, play a critical role in modulating nutrients transfer to the fetus across the placenta. This process is influenced by multiple factors, including placental metabolism, oxidative stress, and blood flow [33,34]. In this study, the gene expression of *GULT1* (one of the key glucose transporters) was increased in the placenta of sows fed the diet with the supplementation of VD_3_, suggesting that VD_3_ might enhance placental glucose transport from the sow to the fetus. Studies have shown that intrauterine growth retardation (IUGR) is strongly associated with placental amino acid transport capacity for protein deposition and fetal tissue growth [35,36,37]. Here, we found that the gene expression of *SNAT2* (one of the main neutral amino acid transporters) in the placenta was accelerated in the 25-OH-D_3_ group, which might explain the higher litter weight of piglets at farrowing in the reproductive performance of sows fed the diet with supplementation of 25-OH-D_3_. Furthermore, it was also found that placental cytokines (*IL-8* and *IL-6*) were decreased in the 25-OH-D_3_ group, indicating that this might repress placental inflammation and inhibit the inflammation response in the maternal–fetal interface at farrowing.

## 5. Conclusions

Our findings indicate that feeding primiparous sows a diet supplemented with 25-OH-D_3_ during mid-to-late gestation resulted in better sow performance. This improvement was likely associated with a reduction in maternal MDA concentrations and the gene expressions of placental *HO-1*, *GPX2*, *IL-6,* and *IL-8* and an increase in the gene expression of amino acid transporter *SNAT2*. These changes helped alleviate oxidative stress and inflammation in both the mother and fetus and enhanced nutrient transport for fetal growth. Additionally, VD_3_ also facilitated maternal antioxidant capacity and placental function. However, no improvement in sow reproductive performance was observed in this study, indicating that 25-OH-D_3_ might be more effective for sow performance than VD_3_ at the same inclusion level.

## Figures and Tables

**Table 1:** Ingredient composition and nutritional levels of the basal diets for pregnant sows (%, as-fed basis).

Ingredients	Composition (%)
Corn	64.65
Soybean meal	16.00
De-fatted rice bran	8.00
Wheat bran	7.00
Soybean oil	1.40
Monocalcium phosphate	0.40
Limestone (CaCO_3_)	1.50
L-Lysine HCl	0.25
Threonine	0.04
Salt	0.40
Choline chloride	0.04
Phytase, 10,000 U/g	0.02
Premix ^1^	0.30
Calculated nutrient content	
Net Energy, kcal/kg	2470
Crude protein, %	14.46
Ca, %	0.69
Total P, %	0.54
Available P, %	0.26
Lysine, %	0.63
Methionine, %	0.24
Threonine, %	0.42
Tryptophan, %	0.14

^1^ Premix supplied per kg of diet: Vitamin A, 25,000 IU; Vitamin D3, 2000 IU; Vitamin E, 37.6 mg; Vitamin K3, 3.2 mg; Vitamin B2, 5.2 mg; Vitamin B6, 2.4 mg; Vitamin B12, 1.2 mg; niacin, 26 mg; pantothenic acid, 21.4 mg; folic acid, 1.2 mg; biotin, 0.18 mg; iron, 80 mg; manganese, 50 mg; copper, 15 mg; selenium, 0.3 mg; iodine, 0.3 mg.

**Table 2:** Primer sequences used for quantitative real-time PCR.

Gene	Primer Sequences	Product Length, bp	Accession No.
CAT	F: CCTGCAACGTTCTGTAAGGC	72	NM_214301.2
	R: GCTTCATCTGGTCACTGGCT		
GAPDH	F: GCTTGTCATCAATGGAAAGG R: CATACGTAGCACCAGCATCA	86	NM_001206359.1
GLUT1	F: CGTCGCTGGCTTCTCCAACTG	110	XM_021096908.1
	R: CCAGGAGCACCGTGAAGATGATG		
GPX1	F: TCTCCAGTGTGTCGCAATGA	104	NM_214201.1
	R: TCGATGGTCAGAAAGCGACG		
GPX2	F: AGCCCCACTGTGAAATTCTT	131	NM_001115136.1
	R: CGTAGAAGGACTTGGCAATG		
HO-1	F: GAGAAGGCTTTAAGCTGGTG	74	NM_001004027.1
	R: GTTGTGCTCAATCTCCTCCT		
IL-1β	F: CAAGGAGATGATAGCAACAA	87	NM_214055.1
	R: CATCACACAAGACAGGTACA		
IL-6	F: AATGTCGAGGCTGTGCAGATT	82	NM_214399
	R: TGGTGGCTTTGTCTGGATTCT		
IL-8	F: CCGTGTCAACATGACTTCCAA	75	NM_213867
	R: GCCTCACAGAGAGCTGCAGAA		
Nrf2	F: GACCTTGGAGTAAGTCGAGA	103	XM_005671981.3
	R: GGAGTTGTTCTTGTCTTTCC		
SOD1	F: GAAGACAGTGTTAGTAACGG	93	NM_001190422.1
	R: CAGCCTTGTGTATTATCTCC		
SOD2	F: GCTGAAAAAGGGTGATGTTA	81	NM_214127.2
	R: CTATGATTGATGTGGCCTCC		
SNAT2	F: GCCGCAGCCGTAGAAGAATGATG	125	NM_001317081.1
	R: AAGCAATTCCGTCTCAACGTGGTC		
SNAT1	F: GCAGGTCTTCGGCACCACAG	80	XM_003355629.4
	R: GGTAGCTCAGCATTGCTCCAGTG		
TNF-α	F: TGGCCCCTTGAGCATCA	68	NM_214022
	R: CGGGCTTATCTGAGGTTTGAGA		
VEGFA	F: CGAGACCCTGGTGGACATCT	115	XM_013977975.1
	R: CTCCAGACCTTCGTCGTTGC		

**Table 3:** The effect of dietary supplementation with VD_3_ and 25-OH-D_3_ on the reproductive performance of primiparous sows during mid-to-late gestation.

Items	CT	VD_3_	25-OH-D_3_	SEM	*p*-Value
Number of sows	15	15	15		
Body weight, kg					
Day 60 of gestation	199	198	198	2.9	0.998
Day 107 of gestation	221	218	222	2.3	0.551
Average backfat thickness, mm					
Day 60 of gestation	18.69	18.99	18.97	0.10	0.071
Day 107 of gestation	19.66	19.73	19.94	0.15	0.603
At farrowing					
Total fetus, n	13.57	14.00	13.86	0.469	0.824
Live fetus, n	12.36	12.93	13.07	0.438	0.507
Stillborn, n	0.64	0.73	0.64	0.245	0.956
Mummy, n	0.50	0.33	0.14	0.186	0.427
Average litter weight, kg	15.58 ^ab^	15.47 ^b^	17.79 ^a^	0.670	0.032
Average birth weight, kg	1.27 ^ab^	1.21 ^b^	1.38 ^a^	0.041	0.015
Percentage of average birth weight < 1.0 kg	12.04	6.13	6.50	10.391	0.244

CT = control group fed with the basal diet; VD_3_ = VD_3_ group fed with the supplementation of 2000 IU/kg Vitamin D_3_ in the basal diet; 25-OH-D_3_ = 25-OH-D_3_ group fed with the supplementation of 50 µg/kg 25-OH-D_3_ in the basal diet. SEM = standard error of the mean (*n* = 15). ^a,b^ Means in the same row with different letters indicate significant differences (*p* < 0.05).

**Table 4:** The effect of dietary supplementation with VD_3_ and 25-OH-D_3_ on the plasma antioxidant capacity of primiparous sows during mid-to-late gestation.

Items	CT	VD_3_	25-OH-D_3_	SEM	*p*-Value
Gestation d60					
SOD, U/mL	12.23	12.23	12.83	0.604	0.761
MDA, nmol/mL	3.41	3.37	3.41	0.306	0.961
GSH-Px, U/mL	751	728	753	44.7	0.911
CAT, U/mL	3.31	3.49	3.11	0.377	0.860
Gestation d107					
SOD, U/mL	13.78	13.22	12.89	0.813	0.780
MDA, nmol/mL	4.41 ^a^	3.28 ^b^	3.37 ^b^	0.160	0.001
GSH-Px, U/mL	829	751	760	45.2	0.416
CAT, U/mL	2.53	2.48	2.79	0.391	0.831

CT = control group fed with the basal diet; VD_3_ = VD_3_ group fed with the supplementation of 2000 IU/kg Vitamin D_3_ in the basal diet; 25-OH-D_3_ = 25-OH-D_3_ group fed with the supplementation of 50 µg/kg 25-OH-D_3_ in the basal diet. SEM = standard error of the mean (*n* = 15). SOD = superoxide dismutase; MDA = malondialdehyde; GSH-Px = glutathione peroxidase; CAT = catalase. ^a,b^ Means in the same row with different letters indicate significant differences (*p* < 0.05).

**Table 5:** The effect of maternal dietary supplementation with VD3 and 25-OH-D3 during mid-to-late gestation on the antioxidant capacity in the umbilical cord blood of newborn piglets.

Items	CT	VD_3_	25-OH-D_3_	SEM	*p*-Value
SOD, U/mL	6.47	6.80	6.24	0.712	0.875
MDA, nmol/mL	7.62	8.24	7.16	0.387	0.239
GSH-Px, U/mL	479	509	527	29.5	0.324
CAT, U/mL	3.07	3.02	2.98	0.275	0.971

CT = control group fed with the basal diet; VD_3_ = VD_3_ group fed with the supplementation of 2000 IU/kg Vitamin D_3_ in the basal diet; 25-OH-D_3_ = 25-OH-D_3_ group fed with the supplementation of 50 µg/kg 25-OH-D_3_ in the basal diet. SEM = standard error of the mean (*n* = 15). SOD = superoxide dismutase; MDA = malondialdehyde; GSH-Px = glutathione peroxidase; CAT = catalase.

## Data Availability

All data are included in the article.
